# Association between Work-Related Musculoskeletal Disorders and Workload among Healthcare Workers

**DOI:** 10.1192/j.eurpsy.2026.11502

**Published:** 2026-07-31

**Authors:** A. Feki, I. Sellami, A. Abbes, A. Haddar, M. L. Masmoudi, M. Hajjeji, K. J. Hammemi

**Affiliations:** 1 Rheumatology Department; 2 Occupationnel Medicine Department, Hedi Chaker Hospital, Sfax, Tunisia

## Abstract

**Introduction:**

Work-related musculoskeletal disorders (WMSDs) represent a major occupational health problem in healthcare professions, affecting productivity, quality of care, and well-being. Healthcare workers are exposed to physical and psychosocial risk factors, including repetitive movements, awkward postures, and high cognitive and emotional demands. The NASA Task Load Index (NASA-TLX) is a validated multidimensional tool that quantifies subjective workload across six domains (mental, physical, temporal demands, performance, effort, and frustration). Understanding the relationship between perceived workload and WMSDs may guide preventive interventions in healthcare settings.

**Objectives:**

This study aimed to evaluate the association between the occurrence of WMSDs and the level of subjective workload measured by NASA-TLX among healthcare personnel, including nurses, nurse assistants, anesthesia technicians and geriatric caregivers.

**Methods:**

A cross-sectional analytical study was conducted among healthcare workers in hospital departments. Participants completed a self-administered questionnaire including sociodemographic data, occupational characteristics, the Nordic Musculoskeletal Questionnaire for WMSDs screening, and the NASA-TLX scale for workload assessment. Statistical analyses were performed using SPSS software. Descriptive statistics summarized prevalence and workload scores, while chi-square tests and logistic regression identified associations between WMSDs and workload dimensions.

**Results:**

Among the participants (n = 146), 28.8 % were males with a mean age of 34.44 ±6,9 years. the overall prevalence of WMSDs during the past 12 months was 75%, with the lumbar (66.4%), cervical (61%), and shoulder (50%) regions being the most affected. Concerning the workload, the highest subscale of NASA-TLX score observed for physical (79±17 /100) and temporal (76,2 ± 25 /100) demands. However, no statistically significant association was found between global or individual NASA-TLX dimensions and the presence of WMSDs (p > 0.05).

**Conclusions:**

Although WMSDs were common among healthcare personnel, this study did not reveal a significant link between subjective workload assessed by NASA-TLX and musculoskeletal complaints. These findings suggest that factors other than perceived workload—such as ergonomic constraints, repetitive movements, and postural strain—may play a more dominant role in WMSD development. Future longitudinal studies are needed to better understand the complex interaction between workload perception and musculoskeletal health in healthcare environments.

**Disclosure of Interest:**

None Declared

